# Specialist Palliative Care Consultation for Patients with Nonmalignant Pulmonary Diseases: A Retrospective Study

**DOI:** 10.1089/pmr.2022.0068

**Published:** 2023-04-14

**Authors:** Hanna Pihlaja, Heidi Rantala, Sirpa Leivo-Korpela, Lauri Lehtimäki, Juho T. Lehto, Reetta P. Piili

**Affiliations:** ^1^ TUNI Palliative Care Research Group, Faculty of Medicine and Health Technology, Tampere University, Tampere, Finland.; ^2^ Palliative Care Centre, Department of Oncology, Tampere University Hospital, Tampere, Finland.; ^3^ Department of Respiratory Medicine, Tampere University Hospital, Tampere, Finland.; ^4^ Palliative Care Centre and Department of Geriatrics, Tampere University Hospital, Tampere, Finland.; ^5^ Allergy Centre, Tampere University Hospital, Tampere, Finland.

**Keywords:** chronic obstructive pulmonary disease, consultation, end-of-life care, interstitial lung disease, nonmalignant pulmonary disease, palliative care

## Abstract

**Background:**

Few patients with chronic nonmalignant pulmonary diseases receive specialist palliative care consultation, despite their high symptom burden in end of life.

**Objectives:**

To study palliative care decision making, survival, and hospital resource usage in patients with nonmalignant pulmonary diseases with or without a specialist palliative care consultation.

**Methods:**

A retrospective chart review of all patients with a chronic nonmalignant pulmonary disease and a palliative care decision (palliative goal of therapy), who were treated in Tampere University Hospital, Finland, between January 1, 2018 and December 31, 2020.

**Results:**

A total of 107 patients were included in the study, 62 (58%) had chronic obstructive pulmonary disease (COPD), and 43 (40%) interstitial lung disease (ILD). Median survival after palliative care decision was shorter in patients with ILD than in patients with COPD (59 vs. 213 days, *p* = 0.004). Involvement of a palliative care specialist in the decision making was not associated with the survival. Patients with COPD who received palliative care consultation visited less often emergency room (73% vs. 100%, *p* = 0.019) and spent fewer days in the hospital (7 vs. 18 days, *p* = 0.007) during the last year of life. When a palliative care specialist attended the decision making, the presence and opinions of the patients were recorded more often, and the patients were more frequently referred to a palliative care pathway.

**Conclusions:**

Specialist palliative care consultation seems to enable better end-of-life care and supports shared decision making for patients with nonmalignant pulmonary diseases. Therefore, palliative care consultations should be utilized in nonmalignant pulmonary diseases preferably before the last days of life.

## Introduction

The need for palliative care has been recognized not only in cancer patients but also in those with many nonmalignant diseases. Patients with chronic progressive pulmonary diseases, such as chronic obstructive pulmonary disease (COPD) or interstitial lung diseases (ILDs), have as high symptom burden in end of life as patients with lung cancer.^1–4^

Already years before death, patients with nonmalignant pulmonary diseases suffer from breathlessness, cough, fatigue, anxiety, depression, and pain, and their quality of life is often markedly decreased.^5–9^

Compared with patients with cancer, a few patients with chronic pulmonary diseases receive palliative care.^10–18^ Cancer patients are also referred to palliative care earlier and their initial palliative care contact is more often an outpatient visit, whereas patients with chronic pulmonary diseases are more likely to have their first palliative care contact in the intensive care unit or during other hospitalization.^4^

Symptom-alleviating medications are less often used in patients with nonmalignant diseases, and they undergo more diagnostic tests and invasive ventilation during the last year of life compared with cancer patients.^12,13,19^ Specialist palliative care may decrease the number of emergency department visits, hospitalization, and end-of-life costs, but the quality of evidence is low and the most effective ways to integrate palliative care in the care of nonmalignant pulmonary diseases remain unknown.^20^

One of the main reasons for the poor integration and late timing of palliative care in nonmalignant pulmonary diseases is the difficulty to define the need for palliative care and appropriate timing for referral to palliative care services.^18^ The Finnish national palliative care guidelines and recommendations of the Ministry of Health and Welfare instruct physicians to recognize patients with a need to shift the goal of the treatment to symptom-centered palliative care by making a palliative care decision.^21,22^

Palliative care decision identifies patients with very advanced and incurable diseases when disease trajectory cannot be markedly influenced by disease-centered treatment options and the intent of the treatment is palliative. This decision is documented by the International Classification of Diseases (ICD)-10 code Z51.5 (Palliative care) in the patient records.

All physicians are recommended to make these decisions when appropriate and to consult or refer the patient to specialist palliative care services if needed. Specialist palliative care consultation services should be arranged in all secondary care hospitals in Finland and are currently available in all five university hospitals, including Tampere University Hospital.

Timely palliative care decisions and palliative care services have been shown to decrease aggressive oncologic therapies, emergency visits, and stays in acute hospitals during the end of life among cancer patients.^23,24^ However, the practice of palliative care decisions and specialist consultations concerning patients with nonmalignant pulmonary diseases has not been described.

The aims of this study were to describe the timing and clinical practice of palliative care decisions in patients with nonmalignant pulmonary diseases and to explore the association of a specialist palliative care consultation on the decision-making process, survival time, and hospital resource usage.

## Materials and Methods

### Design

We conducted a retrospective study of all patients with a nonmalignant pulmonary disease and a palliative care decision treated in Tampere University Hospital from January 1, 2018 to December 31, 2020. Patients were followed up until death or December 31, 2021.

Approval for this study and a permission to access the patient records were obtained from the Pirkanmaa Hospital District, Tampere, Finland (R20592). According to the Finnish law and research regulations, no ethics committee approval was needed for this retrospective register-based study. This study was conducted according to national laws, regulations, and the declaration of Helsinki.

### Setting

Tampere University Hospital provides secondary health care for a population of 530,000 inhabitants of the Pirkanmaa Hospital District and tertiary-level care for a population of 900,000 inhabitants. Annually, over 1500 patients with a documented palliative care decision (ICD-10 code Z51.5) are taken care of in the hospital. Palliative care decision is defined as setting the goal of care to symptom-centered palliative care aimed at maximizing quality of life in patients with very advanced diseases when survival cannot be markedly prolonged with therapy, or the patient does not prefer life-prolonging therapies.

The decision should be discussed with a patient and/or his/her closest ones (mandatory if the patient is not able to communicate) aiming at a shared decision making and mutual understanding.

The hospital's palliative care unit offers consultations through consultation team for all the units and wards in Tampere University Hospital or through outpatient visits. Consultations are provided by physicians with a special competency in palliative medicine. In addition, nurses with special training in palliative care are routinely included in the multidisciplinary team and other professionals when needed. The palliative care unit provides about 1000 inpatient consultations and 1200 outpatient visits a year. Any clinician taking care of a patient can ask for a palliative care consultation if the patient agrees to this.

A regional palliative care pathway has been organized in collaboration with the palliative care unit and the communities of the Pirkanmaa Hospital District. The pathway includes home care teams and community hospital wards or the Pirkanmaa hospice, where patients with a palliative care decision may be admitted without needing a visit in the emergency room (ER).

All the communities have a physician and a nurse responsible for the end-of-life care. Patients need to have a palliative care decision to be included in the pathway and may then be referred to these physicians to ascertain qualified end-of-life care.

### Study population/participants

Altogether, 884 patients with a diagnosis of pulmonary disease and a documented palliative care decision (ICD-10 code Z51.5) were identified from the patient records of the Tampere University Hospital. After excluding patients who had another advanced disease (e.g., cancer) as the primary reason for palliative care decision, 107 patients with chronic nonmalignant pulmonary diseases were included in this study.

### Data collection

All the hospital's electronic patient records were reviewed. Collected data included age, sex, main diagnosis, living conditions, smoking status, pack-years, forced expiratory volume in one second, comorbidities, and the date of death. Patients were considered to need help in activities of daily living if they were supported by a caregiver at home (a family member or home care services) or were permanently accommodated in a nursing home or community hospital. Charlson comorbidity index was calculated for each patient.^25,26^ The main diagnosis was defined as the disease that induced the need for palliative care.

For the last year of life, we recorded all ER visits and acute hospitalization days (i.e., pre-planned follow-up visits were excluded) at the Tampere University Hospital.

Intake in a palliative care pathway was reported if the patient was referred to the local physicians and nurses in the communities and was organized to have preplanned end-of-life care by the home care team, the community hospital, or a hospice.

In addition, details of the palliative care decision were evaluated by recording the date and place of the decision, the specialty of the physician responsible for the decision, the presence of the patient and his/her closest ones, opinions of the patient and his/her closest ones on the decision, concomitant decisions to withhold therapies such as resuscitation or intensive care, and the presence of a palliative care specialist.

### Data analysis

As most of the distributions were non-normal, nonparametric tests were used. The Pearson Chi-square test or Fisher's exact test was used for categorical variables and the Mann–Whitney *U* test for continuous variables. Survival estimations were made by the Kaplan–Meier method with log-rank test.

Statistical significance was set as *p*-value <0.05. Analyses were performed using IBM SPSS Statistics version 27.0 (IBM Corp., Armonk, NY, 2020).

## Results

Patient characteristics are represented in Table 1. Of the 107 patients, 62 (58%) had COPD, and 43 (40%) ILD. Other diagnoses (2 patients) included obliterative bronchiolitis and chronic respiratory insufficiency caused by asthma, obesity-hypoventilation, and embolization. The median age at the time of palliative care decision was 77 years.

**Table 1.** Patient Characteristics

Total number, *n*	107
Male, *n* (%)	57 (53)
Age, median years (range)	77 (60–91)
Main diagnosis, *n* (%)	
COPD	62 (58)
Interstitial lung disease	43 (40)
Other	2 (2)
Needing help with ADL, *n* (%)	90 (84)
Smoking status, *n* (%)	
Never-smoker	25 (23)
Ex-smoker	62 (58)
Current smoker	19 (18)
Unknown	1 (1)
Pack-years, median (range)	40 (2–80)
FEV_1_ (% of predicted), median (range)^a^	44 (12–100)
COPD	30 (12–76)
Interstitial lung disease	65 (30–100)
Other	49 (42–56)
Long-term oxygen therapy one year before death, *n* (%)	51 (55)
Charlson comorbidity index, median (range)	6.0 (2–12)

^a^ Missing value in 10 patients due to loss of data in patient records.

ADL, activities of daily living; COPD, chronic obstructive pulmonary disease; FEV_1_, forced expiratory volume in one second.

**FIG. 1.**
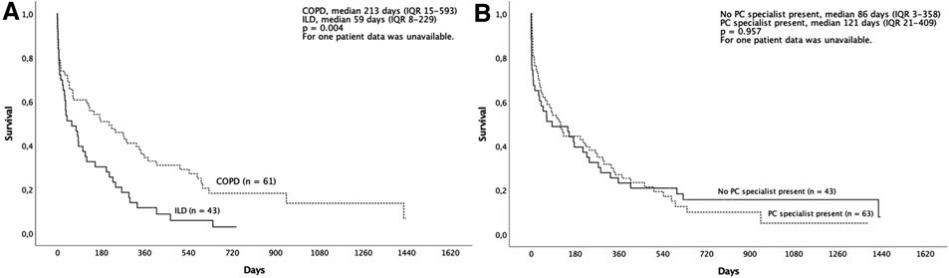
Median survival times after the palliative care decision in patients with COPD or ILD **(A)** and with or without PC consultation **(B)**. COPD, chronic obstructive pulmonary disease; ILD, interstitial lung disease; IQR, interquartile range; PC, palliative care.

Until the end of follow-up, 93 (87%) patients had deceased. The median survival time from the palliative care decision was 118 days (interquartile range [IQR] 11–376). The median survival time after the palliative care decision was significantly longer in patients with COPD (213 days, IQR 15–593) than in those with ILD (59 days, IQR 8–229), (Fig. 1A), whereas the palliative care specialist's involvement in the decision-making process was not associated with the survival time (Fig. 1B).

All the patients had a do not resuscitate (DNR) order recorded. The median time from the DNR order to the palliative care decision was 255 days (IQR 59–676) and to death 479 days (IQR 179–944) in patients with COPD and 18 days (IQR 4–262) and 177 days (IQR 33–453) in patients with ILD, respectively.

Of the deceased patients, 64 (69%) achieved palliative care consultation (Table 2). During the last year of life, the proportion of patients with COPD visiting ER was significantly lower if they received palliative care consultation compared to those without consultation, and they had fewer acute hospitalization days in Tampere University Hospital (Table 2).

**Table 2.** Patients Visiting Emergency Room and Acute Hospitalization Days in Secondary Care Hospital During the Last Year of Life in Patients With or Without Palliative Care Consultation

	Total	Palliative care consultation	No palliative care consultation	***p***
Number of patients, *n* (%)				
All	93	64 (69)	29 (31)	
COPD	51	33 (65)	18 (35)	
ILD	41	30 (73)	11 (27)	
Patients visiting ER, *n* (%)				
Less than one month before death				
All	37 (40)	24 (38)	13 (45)	*0.504*
COPD	21 (41)	13 (39)	8 (44)	*0.726*
ILD	15 (37)	10 (33)	5 (46)	*0.491*
Less than one year before death				
All	75 (81)	48 (75)	27 (93)	*0.041*
COPD	42 (82)	24 (73)	18 (100)	*0.019*
ILD	32 (78)	23 (77)	9 (82)	*1.000*
Number of days spent in hospital, median (range)				
Less than one year before death				
All	10 (0–108)	8 (0–108)	12 (0–61)	*0.209*
COPD	10 (0–93)	7 (0–93)	18 (6–61)	*0.007*
ILD	7 (0–108)	11 (0–108)	3 (0–60)	*0.210*

ER, emergency room; ILD, interstitial lung disease.

The proportion of the patients referred to the palliative care pathway was significantly higher among the patients with than without palliative care consultation (88% vs. 58%, *p* < 0.001). Altogether 17 (18%) patients died in Tampere University Hospital. Of the patients with palliative care consultation, 14% died in the University hospital, compared with 28% of those without a consultation (*p* = 0.118).

Details of the palliative care decisions are shown in Table 3. A palliative care specialist was present during the palliative care decision making in 59% of the cases. This proportion was significantly higher among the patients with ILD than those with COPD (72% vs. 51%, *p* = 0.029). Most of the palliative care decisions were made at hospital wards whether a palliative care specialist was present or not.

**Table 3.** Characteristics of Palliative Care Decisions With and Without the Presence of Palliative Care Specialist

	Total	Palliative care specialist present	No palliative care specialist present	***p***
All patients, *n* (%)	106^a^	63 (59)	43 (41)	
Age, median years (range)		76 (63–91)	78 (60–91)	*0.467*
Disease group, *n* (%)				*0.061*
COPD	61 (58)	31 (49)	30 (70)	
Interstitial lung disease	43 (41)	31 (49)	12 (28)	
Other	2 (2)	1 (2)	1 (2)	
Place of decision, *n* (%)				*0.001*
Ward	64 (60)	40 (64)	24 (56)	
Outpatient clinic	30 (28)	21 (33)	9 (21)	
Intensive care unit	2 (2)	2 (3)	0 (0)	
Other^b^	10 (9)	0 (0)	10 (23)	
Reported as present in decision making, *n* (%)				
Patient	99 (94)	63 (100)	36^c^ (86)	*0.002*
Closest ones	57 (54)	40 (64)	17^c^ (41)	*0.020*
Reported agreement with the decision, *n* (%)				
Patient	82 (78)	55 (87)	27^c^ (64)	*0.005*
Closest ones	35 (33)	24 (38)	11^c^ (26)	*0.205*

^a^ For 1 patient, data were unavailable.

^b^ Decisions made at palliative home care or emergency room.

^c^ For 1 patient, data were unavailable.

When a palliative care specialist attended, patients and/or closest ones were more often reported to be present in the decision making. In addition, patients' agreement to the decision was reported more often if a palliative care specialist was present (*p* = 0.005). If a palliative care specialist attended, decisions to withhold therapies were made more often concomitantly with the palliative care decision, although the difference did not reach statistical significance (43% vs. 26%, *p* = 0.069).

## Discussion

Based on the results of this study, consultations with a palliative care specialist were associated with increased involvement of patients and closest ones in the palliative care decision making and referrals to the palliative care pathway, whereas the consultations were not associated with decreased survival time of the patients. Palliative care consultations also seem to decrease ER visits and days spent in the hospital during the last year of life in patients with COPD. However, these effects are not clear among patients with ILD probably due to the late recognition of the need for palliative care.

In nonmalignant pulmonary diseases, receiving palliative care is associated with better symptom relief and quality of life, increased rate of advance care planning, death outside the hospital, and decreased burden from futile therapies and investigations in end of life.^3,10,18,19,27–30^ The symptom burden and the need of palliative care in different nonmalignant pulmonary diseases are similar but the disease trajectory varies.^31,32^ Patients with COPD may live long with advanced disease and severe breathlessness, whereas survival time is typically shorter among patients with end-stage ILD.^33^

In our study, we also found that survival time was shorter in patients with ILD compared with those with COPD even after the recognition of a need for palliative care decision. Our results are in line with previous studies showing that referral to palliative care and end-of-life care decisions occur late in ILD.^1,34^

This late integration of palliative care in ILD may, at least partly, be due to the fact that although similar signs (e.g., severe dyspnea and functional impairment) of advanced disease are present in all nonmalignant lung diseases, they reflect poorer survival in ILD compared with in COPD, for example. Our results support the current recommendations, that palliative care should be integrated early into disease-modifying treatment options especially in ILD to allow well-timed advance care planning and pre-planned arrangements for end-of-life care.^35^

There is limited evidence on the reduction of hospital resource usage if patients receive specialist palliative care. Some studies show a remarkable decrease in both ER visits and hospital deaths in both COPD and ILD in integrated hospital-based palliative care models.^28,29^ In this study, specialist palliative care consultation was associated with less use of hospital resources during the last year of life in patients with COPD but not in those with ILD.

This finding might be explained by the late recognition of the need of palliative care in ILD as discussed earlier. Nevertheless, we did not find a statistically significant difference in resource usage one month before death in either COPD or in ILD group, probably due to a small sample size. Patients were more often referred to a palliative care pathway if they received palliative care consultation.

This pathway offers pre-planned arrangements for end-of-life care, facilitating easy access to home care services and to end-of-life care in a community hospital ward or a hospice for patients with a palliative care decision. This probably was the major factor explaining the decreased usage of hospital resources through palliative care consultation in our study and highlights the importance of collaboration between in-hospital palliative care teams and regional palliative care providers in the area.

Discussing very advanced diseases and changing the goal of therapy from cure to care (palliative care decision) are delicate issues for patients and their closest ones. When talking about their illness, patients and their closest ones expect honesty, openness, and clarity.^36,37^ In this study, we found aspects reflecting more patient-centered shared decision making when a palliative care specialist was involved in the palliative care decision since patients and their closest ones were reported to be present during the discussion more often and patients' opinion on the decision was also reported more frequently. Similar to a study conducted by Bischoff et al., we found that including a palliative care consultation increased the amount of advance care planning notes and reported decisions to withhold futile therapies.^27^

There is controversial knowledge about the optimal timing of the discussions on goals of care and advance care planning regarding a patient's perspective. Too early discussions may not feel relevant for the patient and there might be hesitance to engage in advance care planning^5,38^ but, on the other hand, in a recent qualitative study, patients expressed willingness to learn about end-of-life regardless of the severity of their disease.^36^

Further, clinicians may also feel uncertain about the optimal timing of these discussions.^39^ In this study, we found that the involvement of a palliative care specialist in the decision-making process is not associated with impaired survival time. This knowledge could encourage pulmonologists to ask for palliative care consultations without a fear of limiting patients' prognosis by, so to say, “giving up” too early.

In patients with nonmalignant pulmonary diseases, palliative care can be integrated to support the physical, emotional, and spiritual well-being of the patients alongside the usual management of the disease itself. Early palliative care and advance care planning could start as early as at the time of the diagnosis of a serious respiratory illness.^35^

This kind of early integration requires collaboration of pulmonary and palliative care clinics to find the best practices to enable consultations. For patients with ILD, a palliative care specialist attended more frequently in the decision making. Shifting of the treatment toward palliative care in ILD may be relatively sudden and occur even during an acute exacerbation with very limited curative treatment options, poor prognosis, and severe symptoms, which may necessitate specialist palliative care intervention from both the clinicians' and patients' perspectives.

Nevertheless, patients with COPD also seem to benefit from a specialist palliative care consultation, and therefore more systematic and early enough consultations in both disease groups are called for.

### Strengths and limitations of the study

This was a real-life study and although there are some limitations, we were able to provide information useful for clinicians taking care of patients with chronic nonmalignant pulmonary diseases. It was possible to retrieve information relevant to this study from the patient records retrospectively.

A relatively small sample size limits the statistical power, which may not have been sufficient to detect weak associations in statistical analyses. Only Tampere University Hospital patient records were evaluated, so we lacked information on the place of death and health care resource usage outside the University Hospital. This is a single-center study from Finland; therefore, the generalizability of the results to other nations and health care systems must be done with caution.

Further prospective studies are needed, which should concentrate on studying these patients through all health care system levels to make more accurate estimations on the effect of a specialist palliative care on resource usage. Qualitative studies are also needed to assess the possible benefits regarding patients' and their closest one's perspectives.

## Conclusions

Our findings suggest that in nonmalignant pulmonary diseases a specialist palliative care consultation allows patients and their closest ones to be better involved in the decision making and leads them more often into a palliative care pathway. Specialist palliative care consultations seem to decrease hospital resource usage during the last year of life, at least in COPD.

The benefits of palliative care consultation in ILD may be impaired by the late recognition of the need of palliative care. Timely advance care planning and integration of specialist palliative consultations to the care of all nonmalignant pulmonary diseases are called for.

## Abbreviations Used

ADL: activities of daily living
COPD: chronic obstructive pulmonary disease
DNR: do not resuscitate
ER: emergency room
FEV_1_: forced expiratory volume in one second.
ICD: International Classification of Diseases
ILD: interstitial lung disease
IQR: interquartile range
PC: palliative care

## References

[1] Rajala K, Lehto JT, Saarinen M, et al. End-of-life care of patients with idiopathic pulmonary fibrosis. BMC Palliat Care 2016;15(1):85; doi: 10.1186/s12904-016-0158-8 27729035 PMC5059981

[2] Carvajalino S, Reigada C, Johnson MJ, et al. Symptom prevalence of patients with fibrotic interstitial lung disease: A systematic literature review. BMC Pulm Med 2018;18(1):78; doi: 10.1186/s12890-018-0651-3 29788938 PMC5964639

[3] Bajwah S, Higginson IJ, Ross JR, et al. Specialist palliative care is more than drugs: A retrospective study of ILD Patients. Lung 2012;190(2):215–220; doi: 10.1007/s00408-011-9355-7 22218887

[4] Wysham NG, Cox CE, Wolf SP, et al. Symptom burden of chronic lung disease compared with lung cancer at time of referral for palliative care consultation. Ann Am Thorac Soc 2015;12(9):1294–1301; doi: 10.1513/AnnalsATS.201503-180OC 26161449

[5] Lindell KO, Kavalieratos D, Gibson KF, et al. The palliative care needs of patients with idiopathic pulmonary fibrosis: A qualitative study of patients and family caregivers. Heart Lung J Acute Crit Care 2017;46(1):24–29; doi: 10.1016/j.hrtlng.2016.10.002 PMC5485906 27871724

[6] Javadzadeh S, Chowienczyk S, Booth S, et al. Comparison of respiratory healthrelated quality of life in patients with intractable breathlessness due to advanced cancer or advanced COPD. BMJ Support Palliat Care 2016;6(1):105–108; doi: 10.1136/bmjspcare-2015-000949 26685116

[7] Rajala K, Lehto JT, Sutinen E, et al. Marked deterioration in the quality of life of patients with idiopathic pulmonary fibrosis during the last two years of life. BMC Pulm Med 2018;18(1):172; doi: 10.1186/s12890-018-0738-x 30458739 PMC6247520

[8] Rantala HA, Leivo-Korpela S, Lehtimäki L, et al. Assessing symptom burden and depression in subjects with chronic respiratory insufficiency. J Palliat Care 2022;37(2):134–141; doi: 10.1177/08258597211049592 34841962 PMC9109583

[9] Seppälä S, Rajala K, Lehto JT, et al. Factor analysis identifies three separate symptom clusters in idiopathic pulmonary fibrosis. ERJ Open Res 2020;6(4):00347-2020; doi: 10.1183/23120541.00347-2020 PMC7533377 33043051

[10] Kraskovsky V, Schneider J, Mador MJ, et al. Longer duration of palliative care in patients with COPD is associated with death outside the hospital. J Palliat Care 2022;37(2):125–133; doi: 10.1177/0825859719851486 31262230

[11] Hyasat K, Sriram KB. Evaluation of the patterns of care provided to patients with COPD compared to patients with lung cancer who died in hospital. Am J Hosp Palliat Med 2016;33(8):717–722; doi: 10.1177/1049909115586395 25987648

[12] Kuo LC, Chen JH, Lee CH, et al. End-of-life health care utilization between chronic obstructive pulmonary disease and lung cancer patients. J Pain Symptom Manage 2019;57(5):933–943; doi: 10.1016/j.jpainsymman.2019.01.011 30708124

[13] Au DH, Udris EM, Fihn SD, et al. Differences in health care utilization at the end of life among patients with chronic obstructive pulmonary disease and patients with lung cancer. Arch Intern Med 2006;166(3):326–331; doi: 10.1001/archinte.166.3.326 16476873

[14] Goodridge D, Lawson J, Duggleby W, et al. Health care utilization of patients with chronic obstructive pulmonary disease and lung cancer in the last 12 months of life. Respir Med 2008;102(6):885–891; doi: 10.1016/j.rmed.2008.01.007 18313278

[15] Butler SJ, Ellerton L, Gershon AS, et al. Comparison of end-of-life care in people with chronic obstructive pulmonary disease or lung cancer: A systematic review. Palliat Med 2020;34(8):1030–1043; doi: 10.1177/0269216320929556 32484762

[16] Koyauchi T, Suzuki Y, Sato K, et al. Quality of dying and death in patients with interstitial lung disease compared with lung cancer: An observational study. Thorax 2021;76(3):248–255; doi: 10.1136/thoraxjnl-2020-215917 33298580

[17] Lindell KO, Liang Z, Hoffman LA, et al. Palliative care and location of death in decedents with idiopathic pulmonary fibrosis. Chest 2015;147(2):423–429; doi: 10.1378/chest.14-1127 25187973 PMC4314817

[18] Zou RH, Nouraie M, Chen X, et al. Assessing patterns of palliative care referral and location of death in patients with idiopathic pulmonary fibrosis: A sixteen-year single-center retrospective cohort study. J Palliat Med 2019;22(5):538–544; doi: 10.1089/jpm.2018.0400 30615545 PMC7869870

[19] Palmer E, Kavanagh E, Visram S, et al. Which factors influence the quality of end-of-life care in interstitial lung disease? A systematic review with narrative synthesis. Palliat Med 2022;36(2):237–253; doi: 10.1177/02692163211059340 34920685 PMC8894683

[20] Bajwah S, Oluyase AO, Yi D, et al. The effectiveness and cost-effectiveness of hospital-based specialist palliative care for adults with advanced illness and their caregivers. Cochrane Database Syst Rev 2020;2020(9):CD012780; doi: 10.1002/14651858.CD012780.pub2 PMC8428758 32996586

[21] Working group set up by the Finnish Medical Society Duodecim, Finnish Association for Palliative Medicine. Palliative and End-of-Life Care. The Finnish Medical Society Duodecim: Helsinki; 2018. Available from: https://www.kaypahoito.fi/en/ccs00037 [Last accessed: August 20, 2022].

[22] Saarto T, Finne-Soveri H; Expert Working Group. Recommendation on the Provision and Improvement of Palliative Care Services in Finland Final Report of the Expert. 2019. Available from: https://julkaisut.valtioneuvosto.fi/handle/10024/161946 [Last accessed: August 20, 2022].

[23] Hirvonen OM, Leskelä RL, Grönholm L, et al. Assessing the utilization of the decision to implement a palliative goal for the treatment of cancer patients during the last year of life at Helsinki University Hospital: A historic cohort study. Acta Oncol (Madr) 2019;58(12):1699–1705; doi: 10.1080/0284186X.2019.1659512 31742490

[24] Hirvonen OM, Leskelä RL, Grönholm L, et al. The impact of the duration of the palliative care period on cancer patients with regard to the use of hospital services and the place of death: A retrospective cohort study. BMC Palliat Care 2020;19(1):37; doi: 10.1186/s12904-020-00547-8 32209075 PMC7093948

[25] Charlson M, Szatrowski TP, Petersoni J, et al. Validation of a combined comorbidity index. J Clin Epidemiol 1994;47(11):1245–1251; doi: 10.1016/0895-4356(94)90129-5 7722560

[26] Charlson ME, Pompei P, Ales KL, et al. A new method of classifying prognostic comorbidity in longitudinal studies: Development and validation. J Chronic Dis 1987;40(5):373–383; doi: 10.1016/0021-9681(87)90171-8 3558716

[27] Bischoff KE, Choi S, Su A, et al. Better together: A mixed-methods study of palliative care co-management for patients with interstitial lung disease. J Palliat Med 2021;24(12):1823–1832; doi: 10.1089/jpm.2020.0787 34115958

[28] Smallwood N, Thompson M, Warrender-Sparkes M, et al. Integrated respiratory and palliative care may improve outcomes in advanced lung disease. ERJ Open Res 2018;4(1):00102-2017; doi: 10.1183/23120541.00102-2017 29707561 PMC5912931

[29] Kalluri M, Claveria F, Ainsley E, et al. Beyond idiopathic pulmonary fibrosis diagnosis: Multidisciplinary care with an early integrated palliative approach is associated with a decrease in acute care utilization and hospital deaths. J Pain Symptom Manage 2018;55(2):420–426; doi: 10.1016/j.jpainsymman.2017.10.016 29101086

[30] Bajwah S, Ross JR, Wells AU, et al. Palliative care for patients with advanced fibrotic lung disease: A randomised controlled phase II and feasibility trial of a community case conference intervention. Thorax 2015;70(9):830–839; doi: 10.1136/thoraxjnl-2014-206583 26103995

[31] Raghu G, Collard HR, Egan JJ, et al. An Official ATS/ERS/JRS/ALAT Statement: Idiopathic pulmonary fibrosis: Evidence-based guidelines for diagnosis and management. Am J Respir Crit Care Med 2011;183(6):788–824; doi: 10.1164/rccm.2009-040GL 21471066 PMC5450933

[32] Traila D, Oancea C, Tudorache E, et al. Clinical profile of unclassifiable interstitial lung disease: Comparison with chronic fibrosing idiopathic interstitial pneumonias. J Int Med Res 2018;46(1):448–456; doi: 10.1177/0300060517719767 28758849 PMC6011274

[33] Rantala HA, Leivo-Korpela S, Lehtimäki L, et al. Predictors of impaired survival in subjects with long-term oxygen therapy. Respir Care 2019;64(11):1401–1409; doi: 10.4187/respcare.06615 30914489

[34] Smallwood N, Mann J, Guo H, et al. Patients with fibrotic interstitial lung disease receive supportive and palliative care just prior to death. Am J Hosp Palliat Med 2021;38(2):154–160; doi: 10.1177/1049909120938629 32648528

[35] Sullivan DR, Iyer AS, Enguidanos S, et al. Palliative care early in the care continuum among patients with serious respiratory illness: An Official ATS/AAHPM/HPNA/SWHPN Policy Statement. Am J Respir Crit Care Med 2022;206(6):e44–e69; doi: 10.1164/rccm.202207-1262ST 36112774 PMC9799127

[36] Kalluri M, Orenstein S, Archibald N, et al. Advance care planning needs in idiopathic pulmonary fibrosis: A qualitative study. Am J Hosp Palliat Med 2022;39(6):641–651; doi: 10.1177/10499091211041724 PMC9082969 34433294

[37] Holland AE, Fiore JF, Goh N, et al. Be honest and help me prepare for the future: What people with interstitial lung disease want from education in pulmonary rehabilitation. Chron Respir Dis 2015;12(2):93–101; doi: 10.1177/1479972315571925 25687210

[38] Hall A, Rowland C, Grande G. How should end-of-life advance care planning discussions be implemented according to patients and informal carers? A qualitative review of reviews. J Pain Symptom Manage 2019;58(2):311–335; doi: 10.1016/j.jpainsymman.2019.04.013 31004772

[39] Gersten RA, Seth B, Arellano L, et al. Provider perspectives on and access to palliative care for patients with interstitial lung disease. Chest 2022;162(2):375–384; doi: 10.1016/j.chest.2022.03.009 35305969 PMC9633804

